# Role of B Lymphocytes in the Pathogenesis of NAFLD: A 2022 Update

**DOI:** 10.3390/ijms232012376

**Published:** 2022-10-16

**Authors:** Chu-Jun Deng, Tak-Ho Lo, Ka-Ying Chan, Xiang Li, Meng-Yao Wu, Zou Xiang, Chi-Ming Wong

**Affiliations:** 1 Department of Health Technology and Informatics, The Hong Kong Polytechnic University, Hong Kong SAR, China; 2 Hong Kong Polytechnic University Shenzhen Research Institute, Shenzhen 518057, China

**Keywords:** non-alcoholic fatty liver disease (NAFLD), non-alcoholic steatohepatitis (NASH), liver fibrosis, B lymphocytes, B cell activating factor, cytokines, autoantibodies

## Abstract

Non-alcoholic fatty liver disease and its related complications are becoming one of the most important health problems globally. The liver functions as both a metabolic and an immune organ. The crosstalk between hepatocytes and intrahepatic immune cells plays a key role in coordinating a dual function of the liver in terms of the protection of the host from antigenic overload as a result of receiving nutrients and gut microbiota antigenic stimulation via facilitating immunologic tolerance. B cells are the most abundant lymphocytes in the liver. The crucial role of intrahepatic B cells in energy metabolism under different immune conditions is now emerging in the literature. The accumulating evidence has demonstrated that the antibodies and cytokines produced by B cells in the microenvironment play key and distinct roles in the pathogenesis of non-alcoholic fatty liver disease (NAFLD). Herein, we have aimed to consolidate and update the current knowledge about the pathophysiological roles of B cells as well as the underlying mechanisms in energy metabolism. Understanding how B cells can exacerbate and suppress liver damage by exploiting the antibodies and cytokines they produce will be of great importance for designing B-cell targeting therapies to treat various liver diseases.

## 1. Introduction

Over-storing fat in the liver through non-alcohol consumption is known as non-alcoholic fatty liver disease (NAFLD). Over-nutrition is one common reason for NAFLD. NAFLD affects up to a quarter of people worldwide [1]. Most people do not have any symptoms. That is the reason why they are not aware that they have NAFLD. Only about 2–5% of people will experience very mild complications such as aching pain in the top right of the tummy and tiredness. If left untreated, the accumulated toxic lipid metabolites lead to liver damage by inflammation and subsequently progress from non-alcoholic fatty liver (NAFL) or simple steatosis to non-alcoholic steatohepatitis (NASH) [2]. One-third of the NASH patients may progress to severe liver fibrosis, cirrhosis, and hence hepatocellular carcinoma [3].

NAFLD is also strongly associated with many common chronic noncommunicable diseases (NCDs), such as type 2 diabetes mellitus, cardiovascular disease, and chronic kidney disease [4]. The clinical burden of these NCDs has been estimated at 85% in industrialized nations. Therefore, it is not difficult to imagine that NAFLD imposes high economic and social burdens, in terms of health-related life quality, work productivity and the use of healthcare resources. Improvements in NAFLD help to resolve metabolic syndrome, cardiovascular disease, and malignancy and vice versa [5]. Unfortunately, NAFLD remains a highly prevalent disease without any approved treatment at this moment [5,6].

## 2. Liver Is Also an Immune Organ

We usually focus on the functions of the liver as a metabolic organ, which is assumed to mainly synthesize and break down various energy-rich metabolites. The metabolic functions of the liver are accomplished by hepatocytes, which make up about 80% of the liver mass. Indeed, the liver is also an immunological organ that contains numerous immune cells specialized in capturing pathogens from circulation, which contributes to the protection of the host from antigenic overload by receiving nutrients and microbiota antigens from the gut via facilitating immunologic tolerance [7]. Tissue-infiltrating immune cells have been shown to regulate liver metabolic functions through paracrine actions of their secreted cytokines, similarly to in other metabolic organs such as adipose tissues [8]. Emerging evidence has demonstrated that hepatic B cells also play a key role in the progression of NAFLD via inflammation. Few mechanisms have been known to provide an explanation as to how B cells promote NAFLD in the obese. Apparently, contradicting findings have also been reported. This review aims to summarize the multiple lines of evidence supporting the roles of B cells in the initiation and exacerbation of NAFLD.

## 3. B Cells and Autoimmune Diseases

B cells are professional antibody-forming cells, which play an important role in adaptive immunity. B cells initiate immune responses through the recognition and interaction of antigens with the B cell receptor (BCR) or through antigen presentation to the helper T cells. It has been proposed that B cells play a key role in autoimmune liver diseases by engaging with autoantigens. The production of autoantibodies by B cells directly contributes to pathogenicity through antibody-mediated cytotoxicity or complement activation, resulting in disorders such as rheumatoid arthritis, systemic lupus erythematosus, and type 1 diabetes [9]. B cells are also capable of producing a large spectrum of cytokines depending on their differentiation and activation states [10]. For example, effector B cells produce Th1-type cytokines, such as interferon (IFN)-γ and interleukin (IL)-12, that promote productive immunity, and regulatory B cells suppress immune responses by producing inhibitory cytokines such as IL-10, IL-35, and transforming growth factor (TGC)-β, and upregulating inhibitory immune checkpoint molecules, such as programmed cell death-1 (PD-1) and programmed death ligand-1 (PD-L1). The ratio of effector to regulatory B cells is critical for maintaining the balance between the productive immunity against pathogens and immune tolerance. Current treatment modalities for autoimmune diseases are focused on the use of immunosuppressive drugs, such as glucocorticoids. The depletion of B cells by rituximab is an alternative approach. However, the long-term use of these drugs in some patients may result in an increased risk of developing hypertension and diabetes [11,12].

## 4. B Cells and Metabolic Dysregulation

Emerging evidence suggests that B cells contribute to the co-morbidities of metabolic diseases, including obesity, diabetes, dyslipidemia, and cardiovascular diseases [13]. The key evidence is that the antibody and cytokine production by B cells is altered in obese subjects [14]. It has been demonstrated that abnormalities in lipid profiles are commonly observed in patients with B cell dysfunction and hyperactivity. For instance, common variable immunodeficiency (CVID) entails decreased high-density lipoprotein (HDL) cholesterol [15,16]. Rheumatoid arthritis (RA) and systemic lupus erythematosus (SLE) are characterized by elevated levels of very low-density lipoprotein cholesterol (VLDL) and triglycerides (TG) [17]. B-cell-depletion therapy based on rituximab (a chimeric monoclonal antibody against CD20) can improve the lipid profile of patients with SLE. Tyrosine kinase inhibitors (e.g., Dastatinib) against the BCR signaling pathway for the treatment of chronic lymphocytic leukemia (CLL) are associated with a worsened glycometabolic profile in patients [18,19]. Taken together, all these findings suggest that B cells play an important role in regulating glucose and lipid metabolism in humans. How do B cells regulate glucose and lipid metabolism?

## 5. B Cells in Liver

B cells represent a major lymphocyte population in the liver. Although the existence of a crosstalk between hepatocytes and immune cells in NAFLD is suggested, research is focused mainly on macrophages [20]. B lymphocytes constitute nearly 6% of intrahepatic cells and represent about 50% of intrahepatic lymphocytes in mice [21]. It is believed that the liver is the graveyard of lymphocytes, where unwanted lymphocytes are trapped and killed [22]. However, significant numbers of intrahepatic B cells do not show any predisposition to apoptosis by using annexin V staining [23]. It has been reported that hepatic stellate cells, by secreting retinoic acid, augment intrahepatic B cell survival and promote their maturation to plasma cells [24].

Interestingly, high-fat high-carbohydrate diet (HFHC) treatment increases the number of intrahepatic B cells in mice, confirmed by flow cytometric analysis [25], and focal aggregates of B cells in the liver of NASH patients have been observed by immunohistochemistry [26]. A recent multi-omics study based on analysis of a total of 111 NAFLD patients from three independent Gene Expression Omnibus (GEO) datasets supports the stratification of NALFD into four distinct molecular subtypes based on gene expression profiles; the redundant infiltration of plasma cells to the liver is a feature in one of the subtypes [27]. Interestingly, it has been revealed that apoptosis of liver-infiltrated B cells is associated with the least severe phenotype of NAFLD [27], supporting the notion of a disease-exacerbating role of B cells in this disorder.

Transcriptomic profiling by single-cell RNA sequencing demonstrates that few intrahepatic B cell clusters isolated from HFHC-fed mice are significantly distinct from their normocaloric diet (NDC) fed littermate controls [25]. What are the key factors triggering the changes in intrahepatic B cells?

## 6. The Role of BAFF in NAFLD

B cell activating factor (BAFF; also known as CD257, BLyS, and TALL-1) is a cytokine that belongs to the tumor necrosis factor (TNF) ligand family. BAFF binds to a number of receptors, including a tumor necrosis factor receptor superfamily member known as BAFF-R or BLyS receptor 3 (BR3), TACI (transmembrane activator and calcium modulator and cyclophilin ligand interactor), and BCMA (B-cell maturation antigen). These receptors are mainly expressed in B cells, which is dependent on the stage of B cell maturation. BAFF stimulates the receptors to regulate B cell maturation and survival, immunoglobulin production, and immunoglobulin class-switch recombination [28,29].

BAFF is mainly produced by many immune cells such as antigen-presenting cells, neutrophils, and activated T lymphocytes. Epithelial cells and adipocytes also produce BAFF [30,31,32]. BAFF is produced as a type II membrane-bound protein and released from cells as a soluble form of BAFF via proteolytic cleavage by the metalloproteases ADAM10 and ADAM17. The circulating BAFF levels are correlated with various immune system disorders such as autoimmune diseases and primary antibody deficiencies [33,34].

Many studies found that increases in BAFF levels are associated with adiposity, insulin resistance, and endothelial dysfunction in overweight and obese subjects. The increased levels of BAFF are also correlated with NAFLD severity [29,35]. Interestingly, the sheddase of BAFF ADAM17 is activated in obese subjects and plays a major regulatory role in obesity, hyperglycemia, hepatosteatosis, and liver inflammation [36,37]. To address the role of B cell activation in the regulation of metabolism, various knockout mouse models are used.

By using a diet-induced obese mouse model, Onji’s team demonstrated that BAFF expression is higher in the white adipose tissues of HFD-fed mice than in their STC-fed littermates [38]. BAFF increases lipid accumulation and induces insulin resistance in adipocytes by increasing the serine 307 phosphorylation and decreasing the tyrosine phosphorylation of IRS-1 in a BAFF-R-dependent manner [38]. Treatment with a BAFF-R FC fusion protein could reverse the BAFF-induced metabolic changes [38]. Indeed, in addition to adipocytes, BAFF-R is expressed in hepatocytes. In contrast, given the fact that BAFF is capable of inducing metabolic changes in adipocytes, it seems that BAFF has a protective role in hepatic steatosis by regulating lipid metabolism in the liver [39]. Hepatic fat deposition has been found to be enhanced in BAFF-R KO mice in the HFD-induced NAFLD model [39]. Microarray analysis to compare gene expression levels between the livers of BAFF-R KO mice and wild-type (WT) mice revealed that the expression of genes associated with fat transportation and biosynthesis is higher in the livers of BAFF-R KO mice than in WT mice [39].

Surprisingly, the depletion of BAFF also attenuates hepatic fat accumulation in a NAFLD mouse model. HFD-fed BAFF KO mice exhibit significantly improved insulin sensitivity despite their increased weight gain and adiposity relative to HFD-fed WT mice [40]. The key changes of HFD-fed BAFF KO mice are characterized by the attenuation of inflammation in adipose tissues through the reduction of macrophage accumulation and the prevention of hepatic steatosis by decreasing de novo lipogenesis in the livers in BAFF KO mice after HFD treatment [40]. Unfortunately, the authors did not check the levels and activities of B cells in their BAFF-KO mice. For example, the common features of BAFF-R deficiencies in humans and mice include very low numbers of circulating B cells, IgM, and IgG antibody levels but increased levels of serum IgA. Furthermore, a high BAFF level increases the risk of developing autoimmunity [41]. It remains to be explored whether B cells contribute to the modulation of the metabolic phenotypes in these models.

## 7. Role of Intrahepatic Regulatory B Cells in NAFLD

**Figure 1:**
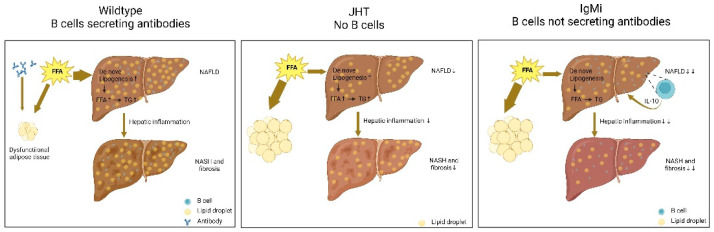
B cells have both detrimental and protective effects in diet-induced NAFLD. The HFD feeding of wild-type mice resulted in manifest NAFLD that was characterized by macrovesicular steatosis associated with massively increased liver weight and a proinflammatory intrahepatic immune response. Both restricted B cell function (IgMi) and complete lack of B cells (JHT) are protected from high-fat diet-induced inflammation and hepatic steatosis. The common feature of JHT and IgMi mice is that they do not secrete antibodies. The pathogenic antibodies secreted by B cells play a significant role in the enhancement of NAFLD pathology by inducing adipose tissue dysfunction. Remarkably, the mice completely lacking B cells (JHT) were partially protected whereas B-cell-harboring but antibody-deficient IgMi mice were completely protected from the development of hepatic steatosis, inflammation, and fibrosis. This is due to the IL-10–producing regulatory B cells providing protection [42].

Recently, Karl et al. used mice with a restricted B cell function (IgMi) or a complete lack of B cells (JHT) to explore the roles of B lymphocytes in NAFLD, established using a diet-induced obesity (DIO) model [42] (Figure 1). The IgMi mouse was firstly introduced in 2007 [43]. IgMi mice have normal B cell development. However, due to the complete deletion of the constant regions in the immunoglobulin heavy (IgH) chain, IgMi mouse can produce cytokines but not any soluble antibodies [44]. The JHT mouse was generated using a Cre-loxP recombination system in 1993 [45]. As the loci for the heavy chain joining region (known as JHT) and the intron enhancer in the IgH locus are deleted, the JHT mouse fails to produce functional B-cells. Both IgMi and JHT mice are protected from high-fat diet-induced weight gain, insulin resistance, and hepatic steatosis [42]. However, IgMi mice attenuate those NAFLD phenotypes to a greater intensity than JHT mice. These data clearly demonstrate that the cytokines produced from B cells may play a key role in the generation of different phenotypes. The authors have concluded that NAFLD is aggravated by pathogenic antibodies but alleviated by cytokines secreted by B cells [42] (Figure 1).

IL-10 is a pleiotropic cytokine with both anti-inflammatory and immunosuppressive effects [46,47]. IL-10 is originally identified as a “cytokine synthesis inhibitor factor” produced by murine T helper 2 (Th2) cells [47]. IL-10 suppresses pro-inflammatory cytokine production by blocking NF-κB activity [48] and regulating the JAK-STAT signaling pathway [49]. Although the serum IL-10 levels of the mouse strain used in the study are not changed in response to HFD treatment, an increase in IL-10 mRNA expression was observed in the liver of HFD-treated IgMi mice, but not JHT mice [42]. Previous studies have shown that a functionally distinct subpopulation of B cells known as regulatory B cells are strong producers of IL-10, which suppresses inflammation [50]. The data from the B cell-deficient mice clearly show that the increase in IL-10 in the liver originates from the intrahepatic regulatory B cells [42]. In addition to IL-10, regulatory B cells also produce many other anti-inflammatory cytokines such as TGF-β and IL-35 [51]. The contribution of these anti-inflammatory cytokines in impeding NAFLD progression remains to be explored.

## 8. Autoantibodies and NAFLD

We have discussed the importance of B cell-derived cytokines in NAFLD in the previous sections. Remarkably, the presence of autoantibodies in NAFLD patients is a general epiphenomenon, and the relationship between the presence of autoantibodies and the severity of NAFLD-related complications has been reported [52,53]. Occasionally, rare autoimmune hepatitis (AIH) may coexist or develop with NAFLD [3]. A recent systematic review and meta-analysis reported that serum antinuclear antibodies (ANAs) can be detected only in approximately one-quarter of subjects with biopsy-proven NAFLD, but it can confer a greater risk of significant fibrosis only in Eastern populations [54].

Previous mouse models transfected with the adenovirus-expressed major liver autoantigen cytochrome P450 2D6 have demonstrated that pre-existing metabolic liver injuries such as NAFLD constitute an additional risk for the severity of an autoimmune condition of the liver [55]. Interestingly, in obese people, decreased B cell activity in response to immunization, such as influenza vaccination, has been observed; however, the significantly increased secretion of IgG autoantibodies is found in these individuals [56]. The same team used in vitro assays with B cells isolated from humans to demonstrate that obesity significantly induces lipid accumulation in B cells, which induces metabolic reprogramming and the expression of the transcription factor T-bet required for autoimmune antibody production in B cells [57]. The next question to answer is how the autoantibodies produced by B cells elicit liver damage by driving pathogenic immune activity.

Albano’s team demonstrated that oxidative stress-derived antigens promoted IgG production in NAFLD by differentiating hepatic B2 cells from IgG-producing plasma cells [58]. They found that patients with NAFLD often show higher circulating levels of antibodies against oxidative stress-derived epitopes (OSEs), and subjects with steatohepatitis have changed their intrahepatic B cell and plasma cell compartments [58]. OSEs are the self-epitopes formed from oxidatively modified endogenous molecules, such as damaged proteins and/or lipoproteins by oxidative stress. The accumulation of OSEs triggers sterile inflammation [59]. Either B2-cell depletion or the intervention with BAFF-mediated survival and the maturation of B2 cells is capable of suppressing the launch of immune responses to OSEs, and hence the evolution of NASH to fibrosis [58].

Another recent example is the finding that autoantibodies against protein disulfide isomerase family A member 3 (PDIA3, also known as ERp57; a protein involved in immunogenic cell death) contribute to the hepatotoxicity of high-fat and high-fructose (HFHF)-fed mice [60]. Mechanically, in brief, lipotoxicity and glucotoxicity are induced by the diet-promoted immunogenic cell death of T and B cells following the MHC-II–restricted presentation of PDIA3 epitopes [60]. Unfortunately, the autoantibodies against these PDIA3 epitopes also target the PDIA3 on the hepatocyte surface and thus exacerbate hepatocyte death, as determined by increased levels of hepatic transaminases in the sera of HFHF-fed mice [60]. Importantly, augmented humoral responses to PDIA3 were also observed in patients with chronic inflammatory liver conditions [60].

In summary, autoantibodies play important roles in the pathogenesis of NAFLD, and their exact impact is based on the type of endogenous proteins that these autoantibodies recognize and the levels of the autoantibodies in the body.

## 9. Roles of B Cells in Fibrosis and HCC

As mentioned above, B cells can enhance the evolution of NASH to fibrosis [58], and anti-BAFF treatment can attenuate NAFLD but not attenuate hepatic fibrosis as measured by collagen deposition, the hepatic expression of collagen-1a, α-smooth muscle actin, and mononuclear cell infiltration in a mouse model [61]. However, the depletion of B cells by anti-CD20 mAb can reduce both NAFLD and hepatic fibrosis [61].

By using carbon tetrachloride (CCl_4_)- and α-naphthylisothiocyanate (ANIT)-induced nonimmune cell–targeted hepatotoxicity in various B-cell–deficient mouse strains, it is demonstrated that B cells are involved in tissue repair and the pathogenesis of liver fibrosis after hepatic injury, but the antibodies are not required for the development of CCl_4_-induced liver fibrosis [23]. Mechanistically, B cells, via the production of fibrogenic TNFα, can contribute to the deposition of collagen derived from hepatic stellate cells (HSC) [62]. Importantly, the presence of infiltrating CD20^+^ B cells correlates with increased tumor aggressiveness and reduced disease-free survival in human hepatocellular carcinoma (HCC) [62,63]. The frequency of plasma cells among total B cells is highest in tumors [63]. The ablation of CD20^+^ B cells by anti-CD20 antibodies in mice promotes senescence-mediated fibrosis resolution and inhibits the protumorigenic TNFα/NF-κB pathway [62]. It is also suggested that the tumor-promoting effect of B cells is mainly attributed to the immunosuppressive function of their IL-10 [64]. Based on these findings, targeting intrahepatic B cell cytokine production, but not antibody production, may be an effective therapy in the treatment of liver fibrosis and HCC.

**Figure 2:**
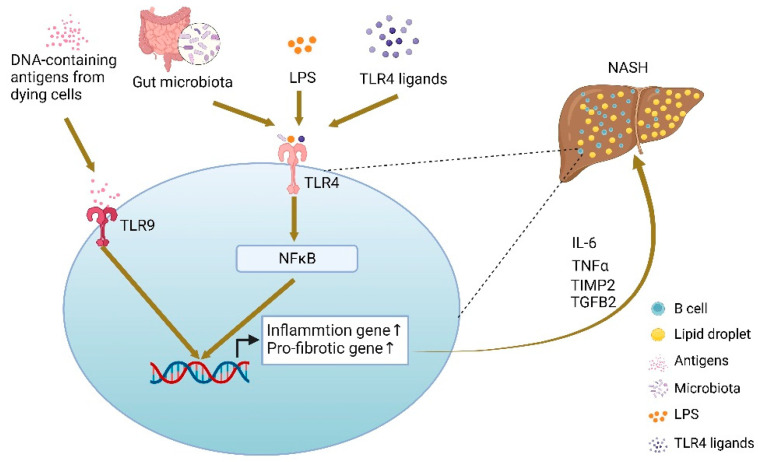
The proposed mechanism of activation of intrahepatic B cells during the disease progression of NASH. B-cell activation during NASH occurs simultaneously through the MyD88 and BCR signaling pathways upon encountering bacteria-derived antigens such as LPS, and bacterial metabolites, as well as through TLR9 which detects endogenous DNA-containing antigens released from dying cells [25], that induces the expression of inflammation and pro-fibrotic genes.

A recent study has also shown that gut microbiota contribute to hepatic inflammation and fibrosis during the disease progression of NASH through the activation of intrahepatic B cells involving both innate and adaptive immune mechanisms [25] (Figure 2). Although it has been generally believed that B cells secrete inflammatory cytokines to a lesser extent when compared with macrophages and neutrophils, by single-cell RNA sequencing, a distinct B-cell population with upregulated inflammatory genes during NASH was identified [25]. This particular group of B cells expresses a high level of BAFF-R. The authors suggest that BAFF may promote the maturation and activation of B cells in NASH [25]. In addition, their RNA-sequencing unveiled that intrahepatic B cells contribute to NASH by expressing the pro-fibrotic genes TGFB1 and TIMP2 [25] (Figure 2). The finding is further supported by the fact that B-cell-deficient mice demonstrate a lower level of fibrosis. Importantly, the restoration of fibrosis can be achieved by reconstitution of B cells isolated from the spleen or liver of HFHC-fed WT donor mice in HFHC-fed B-cell-deficient μMT recipients, suggesting that the development of fibrosis in these HFHC-fed mice is driven by the local activation of B cells, but not other systemic factors [25].

## 10. B Cells and Cholesterol Metabolism

It has been long recognized that cholesterol and its derivatives form critical signaling molecules in regulating immune responses to pathogens or autoimmune diseases via their receptors on various immune cells [65]. Cholesterol induces inflammation, Kupffer cell foaminess, and the formation of the crown-like structure during the pathogenesis of NAFLD [66]. Interestingly, compared with WT mice, the cholesterol levels are significantly decreased in the IgMi and JHT mice [42]. However, the changes in the levels of cholesterol in IgMi and JHT mice are different in terms of HDL and LDL levels. Relatively increased HDL levels are only observed in HFD-fed IgMi mice and increased LDL levels are observed in HFD-fed JHT mice.

Two recent studies independently identified that mutations in *Signal Transducing Adaptor family member 1* [*STAP1*; also known as *BRDG1* (*BCR downstream signaling protein 1*) or *stem cell adaptor protein 1*] are also associated with familial hypercholesterolemia [67,68]. Although the phenotype of *STAP1* mutation carriers is milder than that of *APOB* and *LDLR*, *STAP1* mutation carriers show significantly higher levels of total plasma cholesterol and LDL compared with non-affected relatives [67,68]. However, controversial findings were reported afterward [13,14], and it has recently been proposed to “delist” STAP1 as a hypercholesterolemia gene since many people with mutations in STAP1 do not develop hypercholesterolemia [69]. The controversy might have arisen as a result of the different variants on which these publications have focused (Table 1). As STAP1 is mainly expressed in B cells, it is of great interest to investigate the mutation of STAP1, which will enhance our understanding of the capacity of antibody and cytokine production by B cells in regulating hypercholesterolemia.

## 11. Conclusions and Perspectives

As the diverse roles of intrahepatic B cells in the pathogenesis of NAFLD are observed, further investigations of B cells in this heterogenous liver disease will face more challenges. B-cell-depletion therapies have been adopted for the treatment of autoimmune diseases [76]; however, strategies for further optimizing such cell-depletion-based treatments for B-cell-associated NAFLD and NASH remain to be explored. Different strains of mouse models with manipulated B-cell functionality are valuable tools to further dissect the cellular and molecular mechanisms. The modulation of B cell activation with respect to BCR signaling is considered a more selective therapeutic strategy to ameliorate NAFLD progression with fewer potential side effects than B-cell-depletion therapies [77]. However, as the density of intrahepatic B cells in humans is dramatically lower than in mice [78], the effectiveness of regulating the density and immune status of intrahepatic B cells for controlling the progression of NAFLD in humans remains to be explored.

## Figures and Tables

**Table 1:** List of STAP1 mutations and familial hypercholesterolemia reported previously.

SNPs	Nucleotide	Amino Acid Change	Domain (Position)	Associated with FH	References
rs141647540	c.35G> A	p.Arg12His		No	[70]
				No	[71]
				No	[72]
rs201996284	c.-60A > G	NA		No	[73]
				No	[71]
				No	[72]
rs79388522	c.139A> G	p. Thr47Ala	Pleckstrin homology domain (25-121)	Yes	[68]
rs938523789	c.206T > C	p. Leu69Ser		Yes	[74]
rs141647940	c.212T > C	p. Ile71Thr		Yes	[74]
rs199787258	c.291G > C	p. Glu97Asp		Yes	[74]
				Yes	[72]
				No	[71]
rs14983575	c.414G> C	No changes		No	[70]
rs199787258	c.526C > T	p. Pro176Ser		Yes	[74]
				No	[70]
				No	[71]
rs146545610	c.619G > A	p. Asp207Asn	Src homology 2 domain (177-280)	Yes	[67]
				No	[70]
				No	[75]
rs12948217	c.693C> T	No changes		No	[72]
-	c.803T > C	p. Ile268Thr		No	[73]

## Data Availability

Not applicable.
